# SAMHD1-Deficient CD14+ Cells from Individuals with Aicardi-Goutières Syndrome Are Highly Susceptible to HIV-1 Infection

**DOI:** 10.1371/journal.ppat.1002425

**Published:** 2011-12-08

**Authors:** André Berger, Andreas F. R. Sommer, Jenny Zwarg, Matthias Hamdorf, Karin Welzel, Nicole Esly, Sylvia Panitz, Andreas Reuter, Irene Ramos, Asavari Jatiani, Lubbertus C. F. Mulder, Ana Fernandez-Sesma, Frank Rutsch, Viviana Simon, Renate König, Egbert Flory

**Affiliations:** 1 Division of Medical Biotechnology, Paul-Ehrlich-Institute, Langen, Germany; 2 Research Group “Host-Pathogen Interactions”, Paul-Ehrlich-Institute, Langen, Germany; 3 Division of Allergology, Paul-Ehrlich-Institute, Langen, Germany; 4 Department of Microbiology, Mount Sinai School of Medicine, New York, New York, United States of America; 5 The Global Health and Emerging Pathogens Institute, Mount Sinai School of Medicine, New York, New York, United States of America; 6 Division of Infectious Diseases, Department of Medicine, Mount Sinai School of Medicine, New York, New York, United States of America; 7 Department of General Pediatrics, Münster University Children's Hospital, Münster, Germany; 8 Infectious & Inflammatory Disease Center, Sanford-Burnham Medical Research Institute, La Jolla, California, United States of America; King's College London School of Medicine, United Kingdom

## Abstract

Myeloid blood cells are largely resistant to infection with human immunodeficiency virus type 1 (HIV-1). Recently, it was reported that Vpx from HIV-2/SIVsm facilitates infection of these cells by counteracting the host restriction factor SAMHD1. Here, we independently confirmed that Vpx interacts with SAMHD1 and targets it for ubiquitin-mediated degradation. We found that Vpx-mediated SAMHD1 degradation rendered primary monocytes highly susceptible to HIV-1 infection; Vpx with a T17A mutation, defective for SAMHD1 binding and degradation, did not show this activity. Several single nucleotide polymorphisms in the SAMHD1 gene have been associated with Aicardi-Goutières syndrome (AGS), a very rare and severe autoimmune disease. Primary peripheral blood mononuclear cells (PBMC) from AGS patients homozygous for a nonsense mutation in SAMHD1 (R164X) lacked endogenous SAMHD1 expression and support HIV-1 replication in the absence of exogenous activation. Our results indicate that within PBMC from AGS patients, CD14+ cells were the subpopulation susceptible to HIV-1 infection, whereas cells from healthy donors did not support infection. The monocytic lineage of the infected SAMHD1 -/- cells, in conjunction with mostly undetectable levels of cytokines, chemokines and type I interferon measured prior to infection, indicate that aberrant cellular activation is not the cause for the observed phenotype. Taken together, we propose that SAMHD1 protects primary CD14+ monocytes from HIV-1 infection confirming SAMHD1 as a potent lentiviral restriction factor.

## Author Summary

Lentiviral accessory proteins play important roles in antagonizing host proteins aimed at suppressing HIV-1 replication at a cellular level. The SIV/HIV-2 protein Vpx counteracts SAMHD1, a previously unknown antiviral factor within myeloid blood cells, rendering these cells permissive to primate immunodeficiency viruses. We confirm in this study that Vpx interacts with SAMHD1 leading to ubiquitin-mediated degradation of SAMHD1, and renders CD14 positive monocytes susceptible to HIV-1 infection. We provide new insights into the ability of SAMHD1 to protect monocytic cells from HIV-1 infection by using primary cells from patients with Aicardi-Goutières syndrome (AGS) lacking endogenous SAMHD1 expression. We show that peripheral monocytic cells of AGS patients are highly permissive to HIV-1. Thus, our study demonstrates that SAMHD1 is critical for restriction of HIV-1 infection in monocytes adding SAMHD1 as a novel innate defense factor.

## Introduction

Cells of the myeloid lineage are more refractory to HIV-1 infection than T-cells [1]–[4]. HIV-2 and SIV from sooty mangabeys (SIVsm) but not HIV-1 encode the accessory protein Vpx [5] that provides a replication advantage in human myeloid cells [6], [7]. Moreover, Vpx deficient HIV-2/SIVsm viruses are attenuated *in vivo* [8]. The delivery of Vpx *in trans* through virus-like particles (VLP) also enables HIV-1 to infect otherwise resistant primary human cells such as monocytes [3], [9], [10] or dendritic cells [6], [11]. Furthermore, Vpx promotes HIV infection of macrophages and PMA-differentiated THP-1 cells [12]. Vpx is packaged into budding virions via interaction with the p6 domain of Gag [13] and is active during the early steps of infection in the target cell [5].

Lentiviral accessory proteins counteract known restriction factors such as APOBEC3G or tetherin by mediating their ubiquitin/proteasome-dependent degradation [14], [15]. Similarly, it has been proposed that Vpx allows lentiviral escape by targeting a myeloid cell-specific restriction factor [3], [16], [17] for proteasomal degradation [18]. Two recent publications identified Sterile Alpha Motif (SAM) Domain and HD domain-containing protein 1 (SAMHD1) as the Vpx-sensitive restriction factor that inhibits HIV-1 infection of macrophages and dendritic cells [19], [20].

The *SAMHD1* gene is mutated in a subset of patients suffering from Aicardi-Goutières syndrome (AGS), an early-onset disease that resembles a congenital viral infection [21]. This syndrome is characterized by familial encephalopathy with predominantly neurologic symptoms [22] and increased production of interferon alpha (IFNα) in the brain [23]. Single nucleotide polymorphisms (SNP) in *RNASEH2*, *TREX1* and *SAMHD1* genes have been associated with autoimmunity disorders such as AGS and systematic lupus erythematosus [22]. It has been assumed that the absence of the endonuclease RNASEH2 or the exonuclease TREX1 leads to accumulation of endogenous nucleic acids inducing type I IFN-mediated immune response [24], [25]. In contrast, the role of SAMHD1 in nucleic acid metabolism is not well defined. Moreover, cerebral vasculopathy and strokes accompanied by an altered cytokine secretion pattern have been reported in patients with SNPs in the *SAMHD1* gene [26]–[29].

In this report, in addition to confirming independently the findings by Laguette *et al.* and Hrecka *et al.* [19], [20], we provide further evidence for the role of SAMHD1 as interferon-induced factor restricting HIV-1 replication in monocytes, the progenitors of macrophages and dendritic cells. We demonstrate that SAMHD1 is targeted for ubiquitin-mediated degradation in a Vpx-dependent fashion in primary CD14 positive monocytes. We also found that unstimulated primary peripheral blood mononuclear cells (PBMC) from two AGS patients lacking endogenous SAMHD1 can support viral replication whereas cells from healthy donors encoding wild-type (WT) SAMHD1 were resistant to HIV-1 infection. Microscopy imaging of infected AGS and healthy donor cells suggest that CD14+ cells of monocytic morphology are the cells targeted by HIV-1 in the absence of SAMHD1.

## Results

### SAMHD1 Interacts with Vpx and Is Depleted in a Process that Is Reversible by Addition of MG132

**Figure 1:**
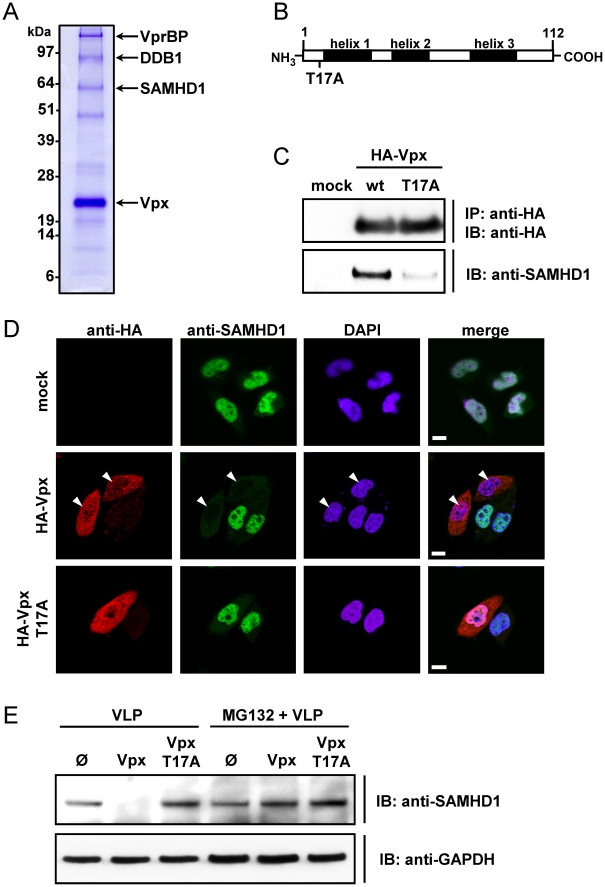
SAMHD1 is identified as Vpx binding protein leading to its degradation. A) pNTAP-SIV Vpx was transfected in 293T cells. After lysis, isolation of SIV-Vpx and binding proteins was performed via Tandem Affinity Purification. The proteins were separated by SDS-PAGE and stained with Coomassie Brilliant Blue. Protein bands in which the respective protein was identified are indicated. B) Schematic representation of the Vpx protein indicating the helical structure elements (black) and the introduced T17A mutation. C) 293T cells were transfected with pcDNA3.1 (mock) or plasmids encoding HA-Vpx or HA-Vpx T17A. After 48 hours, the cells were lysed and subjected to HA-immunoprecipitation. Isolated proteins were separated via SDS-PAGE and analyzed with antibodies specific for HA or SAMHD1. D) HeLa cells endogenously expressing SAMHD1 were transfected with pcDNA3.1 (mock) or HA-Vpx or HA-Vpx T17A encoding plasmids. 24 hours after transfection, the cells were fixed and stained for SAMHD1 and HA-Vpx with fluorescent antibodies. Nuclei were stained with DAPI before performing confocal microscopy. White arrows indicate nuclei of Vpx expressing cells, in which SAMHD1 is absent. White bar length corresponds to 10 µm. E) THP-1 cells were incubated with 2 MOIeq of empty VLPs (Ø), Vpx-VLPs or Vpx T17A-VLPs in the presence or absence of 50 µM MG132. After 6 hours the cells were analyzed by western blot analysis with the indicated antibodies.

To uncover novel Vpx (Vpx_SIVsmPBi_)-interaction partners in 293T cells, tandem affinity purification was performed and isolated proteins were identified by mass spectrometry analysis (Figure 1A and S1). In addition to confirmed Vpx interacting proteins such as VprBP [30] and DDB1 [17] we isolated a 72 kDa protein, which was determined to be SAM domain and HD domain containing protein 1 (SAMHD1). In order to confirm the protein interaction, endogenous SAMHD1 was co-immunoprecipitated with HA-Vpx_SIVsmPBi_, whereas interaction with an inactive Vpx mutant, T17A (Figure 1B) [6], [12], was significantly reduced (Figure 1C). The functional relevance of the SAMHD1-Vpx interaction on a single cell level was further determined by confocal fluorescence microscopy. We observed that expression of wild-type (WT) Vpx leads to a drastic reduction of endogenous nuclear SAMHD1 levels (Figure 1D). Of note, Vpx T17A displayed a cellular distribution similar to that of its WT counterpart (both in nuclei and cytoplasm), but was unable to deplete SAMHD1 (Figure 1D).

Next we probed for the importance of ubiquitin-conjugated protein degradation in the Vpx-mediated reduction of SAMHD1. We observed that the peptide aldehyde proteasome inhibitor MG132 blocked Vpx-mediated depletion of SAMHD1 in THP-1 cells (Figure 1E). The Vpx mutant T17A failed to induce degradation of SAMHD1 (Figure 1E).

Our findings are in agreement with the recent reports of Laguette *et al.* [20] and Hrecka *et al.* [19] that identified SAMHD1 as a Vpx_SIVmac251_ and Vpx_HIV2_ interacting protein. Taken together, these results demonstrate that Vpx targets SAMHD1 for ubiquitin-mediated degradation.

### SAMHD1 Restricts HIV-1 Infection in Monocytic Cells

**Figure 2:**
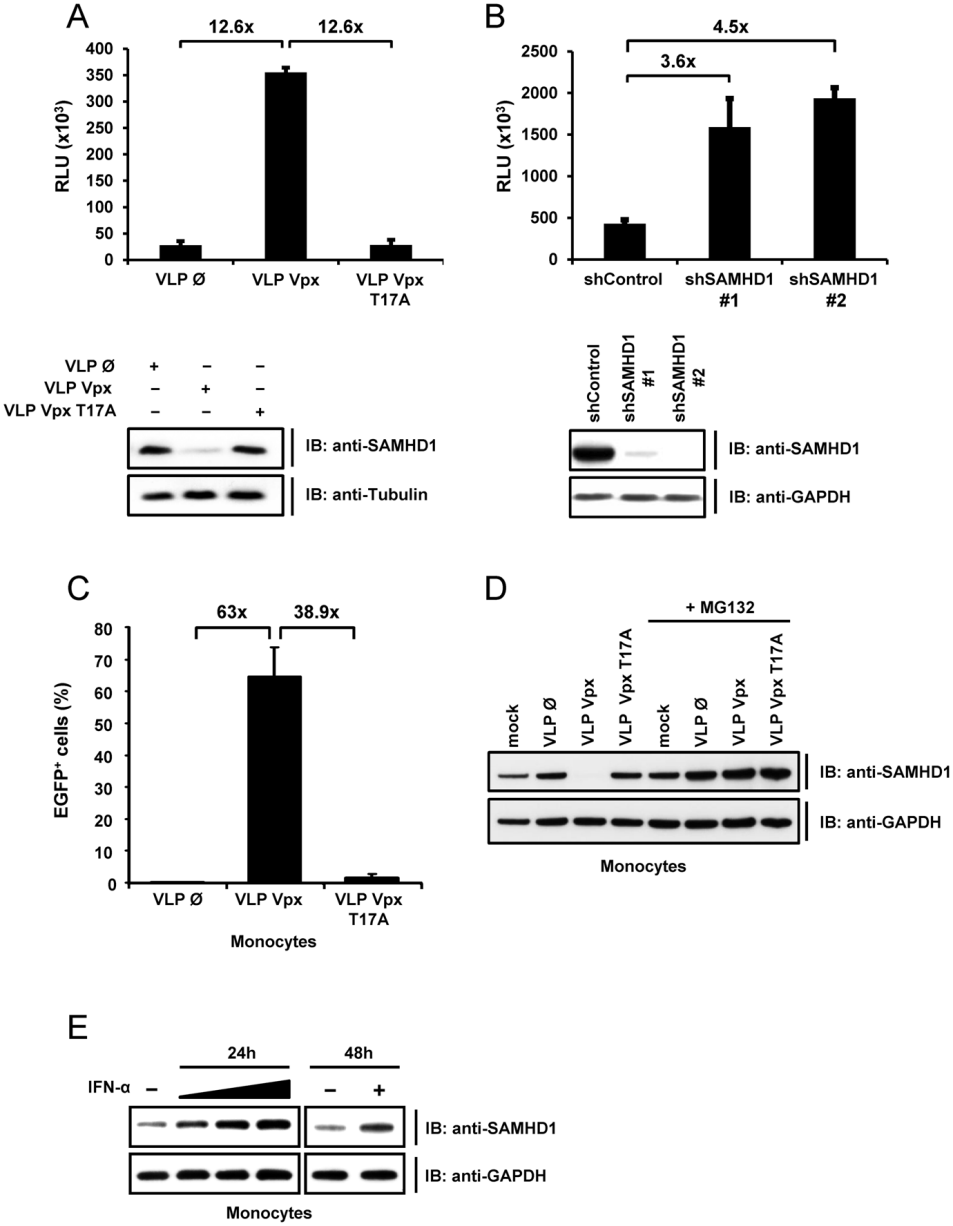
SAMHD1 is a novel interferon-induced restriction factor for HIV-1 in monocytic cells. A) THP-1 cells were stimulated with PMA for 24 hours before they were transduced with empty VLPs (Ø) or VLPs carrying Vpx (VLP VPX) or Vpx T17A mutant (VLP Vpx T17A) with a MOIeq of 2. Two hours after transduction the cells were infected with HIV-1-luc. Luciferase activity was determined 24 hours after infection. In parallel, uninfected THP-1 cells were lysed six hours after VLP transduction and subjected to western blot analysis with the indicated antibodies (lower panel). B) THP-1 cells were transduced with lentiviral particles encoding for a non-specific shRNA (shControl) or a shRNA against SAMHD1 (shSAMHD1 #1; #2) and selected with puromycin. PMA stimulated THP-1 shControl or THP-1 shSAMHD1 (#1; #2) cells were infected with HIV-1-luc. After 24 hours, the luciferase activity was determined. In parallel, uninfected cells were subjected to western blot analysis using the indicated antibodies (lower panel). C) One day post-isolation, primary human monocytes were incubated with 2 MOIeq of VLPs containing no Vpx, WT Vpx or Vpx T17A and HIV-1-EGFP (MOI 8). EGFP expression was determined by FACS five days post infection. The experiment was repeated twice with different donors and the results of a representative experiment are shown here. D) Primary human monocytes were transduced with 2 MOIeq empty VLPs, Vpx VLPs or Vpx T17A VLPs and cultured w/o MG132 (50 µM) for 6 hours. The cells were lysed and analysed by immunoblot for expression of SAMHD1 and GAPDH. Mock indicates untransduced cells. E) Primary human monocytes were stimulated with 2, 20 or 200 ng/ml IFNα (ProSpec) for 24 hours or with 20 ng/ml IFN (Sigma) for 48 hours. The cells were lysed and SAMHD1 was detected with the respective antibody in western blot analysis.

We next tested the hypothesis that the efficiency of HIV-1 infection in monocytic cells correlates directly with Vpx-mediated degradation of SAMHD1. We transduced PMA-differentiated THP-1 cells with VLPs carrying WT Vpx, the Vpx T17A mutant or empty VLPs and subsequently infected them with a single-cycle VSV-G pseudotyped HIV-1 luciferase reporter virus (HIV-1-luc). We found that HIV-1 infection was 12.6-fold higher in the presence of WT Vpx, compared to mutant Vpx or empty particles (Figure 2A), and this phenotype correlated with the degradation of SAMHD1 in these cells (Figure 2A, lower panel).

To test the efficiency of HIV-1 restriction by endogenous SAMHD1, we infected differentiated THP-1 cells stably expressing shRNA targeting SAMHD1 or a non-targeting control shRNA with HIV-1-luc (Figure 2B, lower panel). Infection with single-round HIV-1-luc was up to 4.5-fold higher in SAMHD1 shRNA expressing cells than in control cells (Figure 2B).

Next, we assessed whether Vpx-mediated HIV-1 infection also affects SAMHD1 levels in primary monocytes. Therefore, monocytes were infected with a lentiviral HIV vector expressing GFP (HIV-1-EGFP) in the presence or absence of Vpx. In line with the experiments in THP-1 cells (Figure 2A), delivery of Vpx relieved restriction of HIV-1-EGFP whereas Vpx T17A did not render these cells susceptible for HIV-1 (Figure 2C). Also in monocytes, the permissiveness to HIV-1 correlated with the Vpx-induced reduction of SAMHD1 in a ubiquitin-dependent pathway (Figure 2D). We conclude that monocytes express SAMHD1 as an interferon-inducible factor (Figure 2E) that is degraded upon Vpx-supported HIV-1 infection confirming the results in dendritic cells [20] and macrophages [19].

### Cells from Aicardi-Goutières Syndrome Patients Lacking SAMHD1 Support Spreading Infection of HIV-1

**Figure 3:**
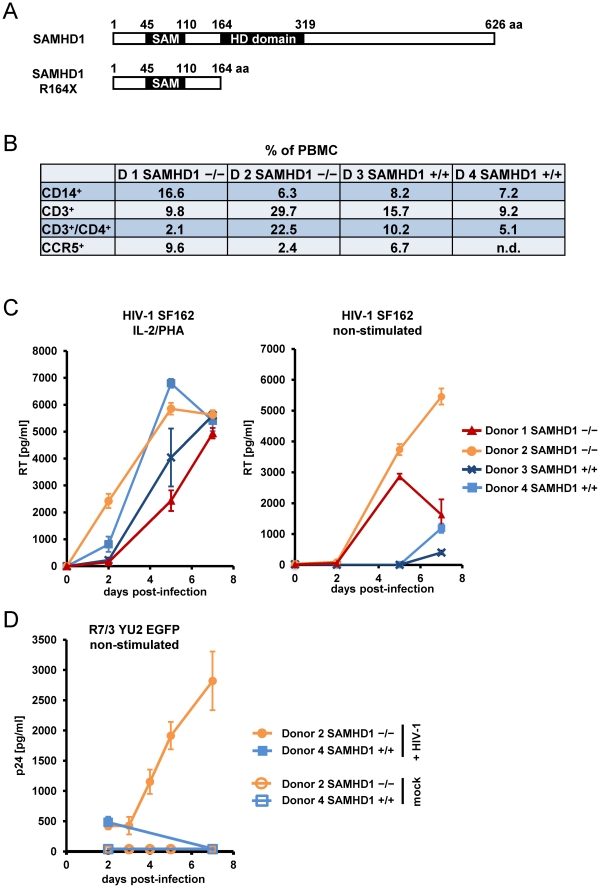
PBMC from AIcardi-Goutières syndrome patients homozygous for SAMHD1 mutation are highly susceptible for spreading HIV-1 replication. A) Schematic representation and domain structure of SAMHD1 and SAMHD1 R164X, indicating the truncation of SAMHD1 after insertion of a STOP codon observed in two AGS patients. B) PBMC from healthy donors (Donor 3/4 +/+) and AGS patients homozygous for SAMHD1 R164X (Donor 1/2 -/-) were isolated by Ficoll gradient, fixed and stained with PE-labeled anti-CD14, PE-labeled anti-CCR5, FITC-labeled anti-CD3 and PE-labeled anti-CD4 for FACS analysis. The numbers indicate the relative amount of the respective subpopulation within PBMC. C) A fraction of isolated PBMC described in (B) was stimulated with 250 ng/ml PHA & 180 U/ml IL-2 for 18 hours (left graph) or were left non-stimulated (right graph) prior to infection in triplicate with 0.05 MOI of HIV-1 SF162. At indicated days post-infection, the supernatants were analyzed for reverse transcriptase (RT) activity. D) A fraction of isolated Donor 2 (SAMHD1 -/-) and Donor 4 (SAMHD1 +/+) PBMC analyzed in (B) were infected in triplicate with HIV R7/3 YU2 EGFP or mock infected. At indicated days post-infection, the culture supernatants were analyzed for p24 concentration by ELISA.

To determine whether cells with a nonsense mutation in SAMHD1 obtained from AGS patients might be more susceptible to viral infection, we tested HIV-1 infectivity in PBMC from two related AGS patients described previously [28]. The cause of AGS in these patients was assigned to an homozygous mutation in SAMHD1, which introduces a premature stop codon at position 164. This results in a truncated protein lacking the HD domain (Figure 3A). The composition of PBMC subpopulations from AGS patients showed no obvious SAMHD1-dependent deviation from PBMC of healthy donors (Figure 3B). PBMC from patients homozygous for SAMHD1 R164X as well as WT SAMHD1 were stimulated with interleukin 2 (IL-2) and PHA followed by subsequent infection with the replication-competent CCR5-tropic HIV-1 SF162. Interestingly, HIV-1 spread in both healthy and AGS primary cells to a comparable degree (Figure 3C, left panel). Western blot analysis of infected PBMC confirmed the absence of full-length, but also truncated SAMHD1 in AGS patients (Figure S2), suggesting that the R164X mutation reduces protein stability or induces nonsense-mediated decay of the transcript. To exclude the impact of mitogen-stimulated T-lymphocytes on HIV-1 replication that might conceal the restrictive role of SAMHD1, we repeated this experiment in the absence of IL-2/PHA stimulation. In non-stimulated cells, HIV-1 spread far more rapidly in SAMHD1 R164X PBMC than in PBMC from healthy individuals (Figure 3C, right panel). These findings suggest that, in the absence of exogenous stimulation, SAMHD1 deficiency renders a subpopulation of cells within PBMC susceptible to HIV-1 replication. However, upon mitogenic T-cell stimulation, the absence of SAMHD1 has no further advantage for HIV-1 replication likely because the bulk of the viral replication occurs in activated T-lymphocytes masking the putative replication in less abundant cell populations.

To confirm the SAMHD1-dependent effect of HIV-1 replication in non-stimulated PBMC, we infected AGS and healthy donor PBMC with R7/3-YU2-EGFP, a CCR5-tropic HIV virus that encodes GFP in place of Nef [31], [32]. However, due to the limited availability of clinical specimen from AGS patients, we could perform these experiments only with PBMC from one of the two AGS patients (Donor 2). Despite low viral input (MOI: 0.01) we observed also in this experimental setup, a sustained replication in non-stimulated AGS PBMC starting as early as day 3 after infection (Figure 3D). No p24 production was observed in PBMC from the healthy Donor 4 within seven days of infection (Figure 3D).

These results indicate that the absence of SAMHD1 due to the R164X mutation supports a spreading replication of HIV-1 in non-stimulated primary cells normally refractory to HIV-1.

### HIV-1 Predominantly Infects CD14+ Monocytes in SAMHD1-deficient PBMC

**Figure 4:**
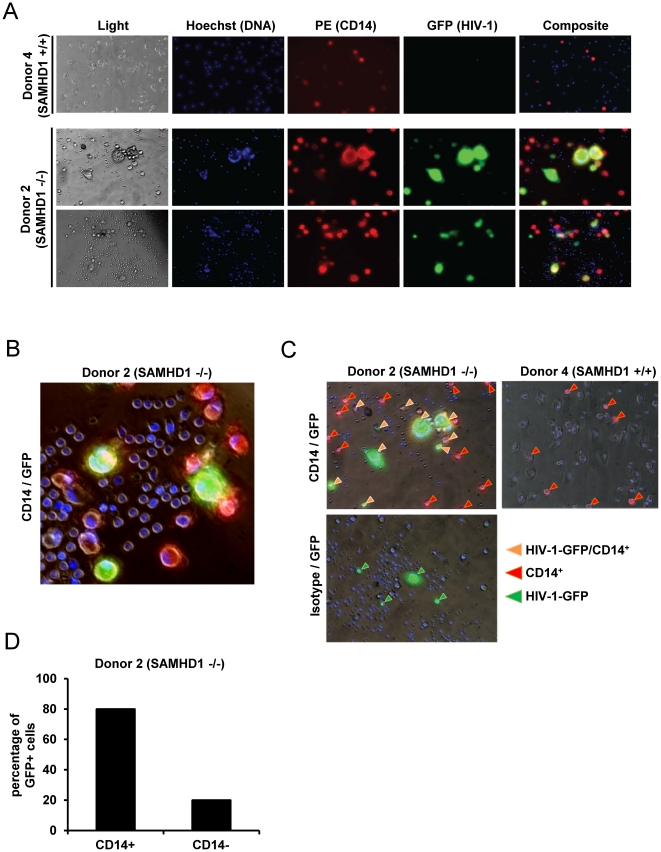
Monocytic cells lacking SAMHD1 are target cells for HIV-1. A) PBMC of the SAMHD1-deficient Donor 2 and the healthy Donor 4 were infected in triplicate with HIV-1 R7/3 YU2 EGFP as described in Figure 3D. After 7 days in culture, staining was performed with CD14-PE (red) and Hoechst 33342 (DNA staining, blue). GFP expression (green) indicates HIV infected cells. Images were acquired with 20x and 40x lenses on an Olympus microscope with live imaging system. B) Magnified selected composite image from infected cells derived from Donor 2 (SAMHD1 -/-). C) Magnified selected composite images from infected cells derived from Donor 2 (SAMHD1 -/-) and Donor 4 (SAMHD1 +/+). Arrows identify CD14+ (red), GFP/HIV+ (green) and CD14+/GFP/HIV-1 + cells (orange). D) Percentage of CD14+ and CD14- cells within HIV-1/GFP+ cells from Donor 2 (SAMHD1 -/-) determined by cell counting of 16 independent microscopy images.

Next, we sought to further define the primary cell population infected with R7/3-YU2-EGFP. The infections of PBMC from Donor 2 (SAMHD1 -/-) and healthy Donor 4 (SAMHD1 +/+) were inspected by live cell fluorescence microscopy at day 7. In agreement with the replication data (Figure 3D), 10% of SAMHD1 -/- cells (Donor 2) were GFP/HIV-1 positive at day 7 post infection whereas PBMC from Donor 4 did not yield green fluorescent cells at any time point (Figure 4A).

Because most of the HIV-1 positive cells were much larger than T-lymphocytes and exhibited a morphology similar to myeloid cells, we stained the cells for the myeloid cell marker CD14 at day 7 post infection in order to determine the susceptibility of this lineage to HIV-1. Counting the cells represented in independent microscopic images (Donor 2: 16 images and Donor 4: 12 images) revealed that within the SAMHD1-deficient specimen 80% of GFP/HIV-1+ cells were positive for CD14 (see Figure 4B and 4C for examples and Figure 4D for quantification). Interestingly, at day 7 of the experiment, the overall percentage of CD14+ cells was higher in the AGS sample (Donor 2: 26% HIV-1 infected, 34% mock infected versus Donor 4: 7% and 8% respectively; 12 independent images; Figure S3), although the frequency of CD14+ cells post isolation were comparable between Donor 2 and Donor 4 (Figure 3B). In summary, these live cell microscopy experiments support the notion that within non-stimulated PBMC lacking functional SAMHD1, CD14+ monocytic cells are the subpopulation that are highly susceptible to HIV-1 infection.

### Activation of SAMHD1 -/- and SAMHD1 +/+ Cells before and after HIV-1 Infection

**Figure 5:**
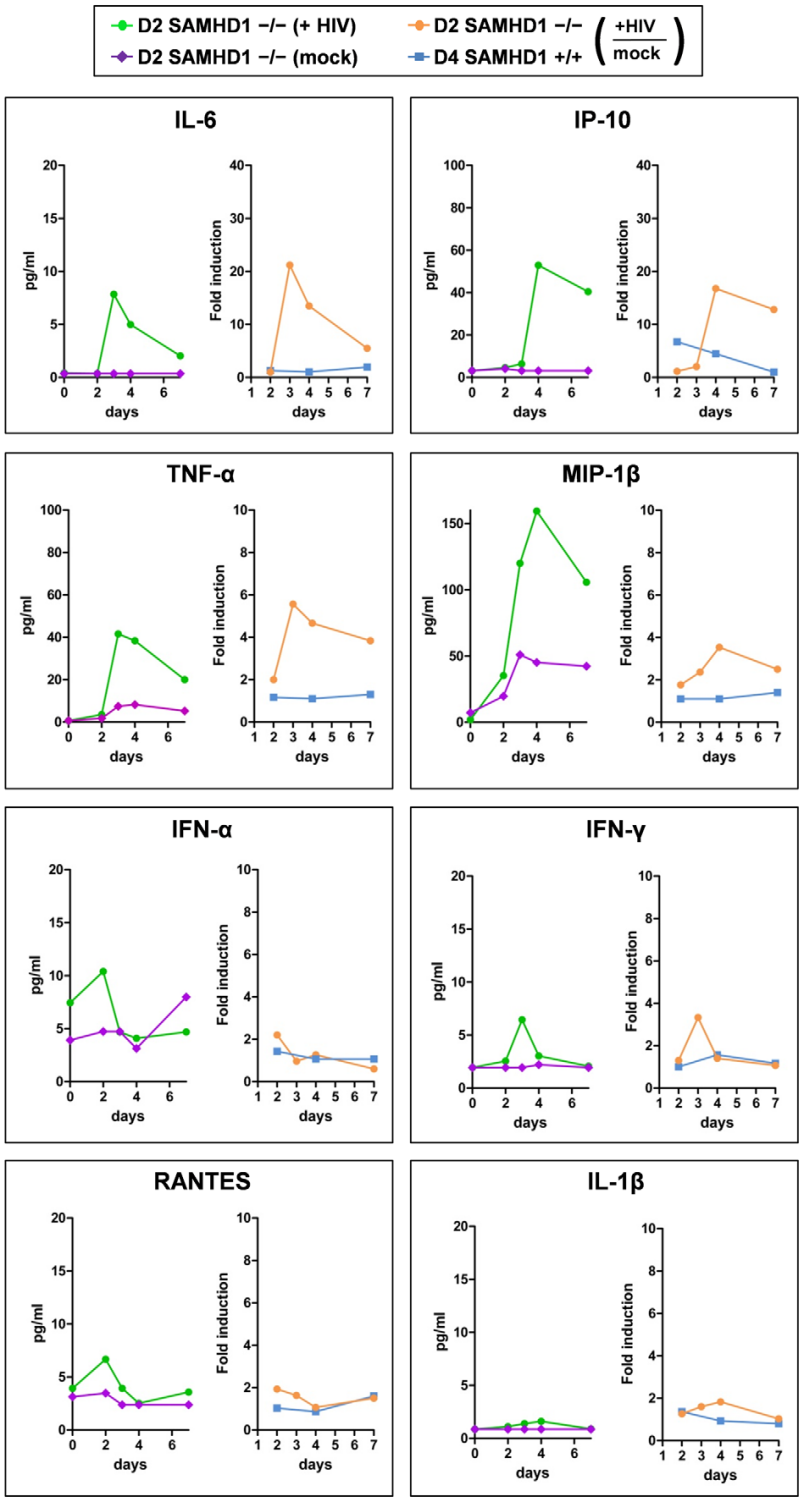
Cytokine secretion of PBMC lacking SAMHD1 indicates an early inflammatory response during HIV-1 replication. The culture supernatants of HIV-1 R7/3 YU2 EGFP infected and non-infected PBMC of the SAMHD1-deficient Donor 2 and the healthy Donor 4 as described in Figure 3D were subjected to Multiplex ELISA and analyzed for time-dependent secretion of the indicated chemokines and cytokines. The results are presented as cytokine concentrations for SAMHD1-deficient Donor 2 in the absence (purple lines) or presence (green lines) of HIV-1 infection and as fold induction upon HIV-1 infection over the mock infected controls for SAMHD1-deficient Donor 2 (yellow lines) and healthy Donor 4 (blue lines).

To determine the interferon and cytokine/chemokine responses during HIV-1 replication, we analyzed the culture supernatants of R7/3-YU2-EGFP or non-infected cells from Donor 2 (SAMHD1 -/-) and Donor 4 (SAMHD1 +/+) using multiplex-ELISA. Prior to infection, cytokine and chemokine levels were low or undetectable in Donor 2, suggesting that the cells were not activated due to the absence of SAMHD1 (Figure 5, purple line).

Only in AGS PBMC, early pro-inflammatory cytokines such as IL-6, IP-10, TNFα and MIP-1β were induced during the seven day course of HIV-1 replication (Figure 5, green and orange lines). IL-6 and IP-10 were stimulated more than 20-fold at day 3 and more than 15-fold at day 4, respectively, compared to the low level induction in healthy donor 4, suggesting an early inflammatory reaction upon HIV-1 in SAMHD1-deficient PBMC (Figure 5, orange and blue lines). These cytokines are also indicative of a cytokine response primarily produced by cells of monocytic origin [33], [34]. Notably, IFN-α, IFN-γ, the CCR5-ligand RANTES and the cytokine IL-1β were neither induced upon HIV-1 replication in SAMHD1-deficient cells nor upon HIV-1 incubation with healthy donor PBMC (Figure 5). We also could not detect evidence for secretion of bioactive type I interferon using a sensitive interferon bioassay (Figure S4).

## Discussion

In this study, we determine the role of SAMHD1 as a HIV-1 restriction factor in human monocytes confirming and extending the findings observed in other myeloid cells [19], [20]. Previous reports have determined that residue 17 in Vpx is crucial for the infection of macrophages and dendritic cells [6], [12]. Our results, using a Vpx T17A mutant, explain this observation by revealing that the Vpx-SAMHD1 interaction most probably occurs via the region encompassing Vpx residue 17. Moreover, we confirm the effect of the proteasome inhibitor MG132 on the reversibility of Vpx-mediated degradation of SAMHD1 [19], [20]. MG132 inhibition of the proteasome can also have the effect of depleting cellular ubiquitin levels and, thus, block any ubiquitin-dependent processes [35]. Vpx-mediated SAMHD1 depletion may, therefore, be due to proteasomal degradation or other ubiquitin-dependent mechanisms (e.g., ubiquitin dependent recruitment to the lysosome).

SAMHD1 consists of a sterile alpha motif (SAM), that can serve as a protein-binding [36] or RNA-binding domain [37] and a HD domain characterized by a doublet of histidine/aspartic acid residues. The latter is presumed to bind nucleic acids [38] and to serve as a phosphohydrolase/nuclease domain [39], [40]. As with other HIV-1 restriction factors such as Tetherin, TRIM5α and APOBEC3G that are up-regulated by interferon [41], [42], we find that SAMHD1 is regulated by type I IFN. This is consistent with earlier reports suggesting that the host molecule targeted by Vpx is inducible by type I interferon, which was reported to magnify the Vpx phenotype on HIV-1 infection in macrophages [12]. Besides SAMHD1, the myeloid-specific protein APOBEC3A was reported to be counteracted by Vpx [9], [43], suggesting that either protein might act as co-factor for antiviral activity.

Expression of Vpx or silencing of SAMHD1 was reported to induce viral DNA accumulation [3], [20], suggesting that the restriction takes effect at or before reverse transcription [44]. Since SAMHD1 is located in the nucleus (see Figure 1D and [20], [25]), it either might act on components of the pre-integration complex after nuclear entry or it is exported to the cytoplasm during the early phases of infection.

Most interestingly, we observe a spreading replication of HIV-1 in non-stimulated PBMC from AGS patients homozygous for SAMHD1 R164X. Although one should take into account that cells of only one AGS patient were analyzed, our data suggest that predominantly cells of the CD14 positive monocytic lineage became highly susceptible targets for HIV-1 compared to HIV-1 resistant cells of healthy donors. The infected patient cells displayed an early inflammatory cytokine secretion profile that might be interpreted as a monocytic cell specific response to HIV-1 infection. We conclude therefore that SAMHD1 protects monocytic cells from HIV-1 infection. Upon T-cell stimulation in PBMC, the enabled HIV-1 replication did not additionally benefit from SAMHD1 deficiency, allowing the speculation that SAMHD1 might not contribute to HIV-1 restriction in T-cells. However, in this context other restriction-associated cellular determinants and cell type-specific SAMHD1 expression level have to be considered in future studies.

In conclusion, our study sheds light on how HIV-1 and the host's antiviral innate immune responses intersect. Understanding cell-type specific barriers to HIV-1 infection such as mediated by SAMHD1 will be important to develop innovative therapies and vaccines.

## Materials and Methods

### Ethics Statement

Buffy-coats obtained from anonymous blood donors were purchased from the German blood donation center. Whole blood was obtained from AGS patients that signed an informed consent. The research has been approved by the Ethics Committee of the Chamber of Physicians Westfalen-Lippe and the Medical Faculty of the Westfalian Wilhelms University Münster (Reference No 2006-556-f-S) and performed according to the principles expressed in the Declaration of Helsinki.

### Tandem Affinity Purification and Mass Spectrometry

Codon-optimized SIV Vpx from SIVsm PBj1.9 was cloned into pNTAP of the Interplay Mammalian TAP System (Stratagene). 293T cells were transfected and cultivated for 48 hours. One hour before lysis, cells were incubated with 100 ng/ml PMA. Purification was performed as described in the manufacturer's instructions. Proteins associated with the Calmodulin-binding beads were subjected to SDS-PAGE, stained with Coomassie brilliant blue, digested with trypsin and analyzed by tandem mass spectrometry (MS/MS) at the Paul-Ehrlich-Institute.

### Plasmids

Codon-optimized N-terminal HA-tagged SIV Vpx derived from SIVsm PBj1.9 [9] and N-terminal FLAG-tagged SAMHD1 were cloned into pcDNA3.1. The Vpx T17A mutant was generated using the Site-Directed Mutagenesis Kit (Stratagene). Codon-optimized SIV Vpx from SIVsm PBj1.9 was cloned into pNTAP of the Interplay Mammalian TAP System (Stratagene).

### Cell Culture and Primary Cell Isolation

HEK 293T and HeLa cells were grown in Dulbecco's modified Eagle's medium, THP-1 cells were grown in RPMI medium, both containing 1 mM L-glutamine and 10% fetal calf serum (FCS). Anonymized buffy coats were purchased from the German blood donation center for isolation of primary human monocytes with the Monocyte Isolation Kit II (Miltenyi) and the isolated cells were cultured as described previously [9]. For stimulation, monocytes were incubated with interferon α (Sigma # I4784 & ProSpec # Cyt204B).

PBMC were isolated using a Ficoll gradient from whole blood obtained from healthy donors and AGS patients that signed an informed consent. Of note, AGS patient Donor 1, but not Donor 2 was treated with immunosuppressive medication at time of blood collection. PBMC were cultivated in RPMI, Pen/Strep, and 20% FCS.

### Viral Particle Production, THP-1 and Monocyte Transduction

HEK 293T cells were co-transfected with the SIV PBj1.9-derived packaging construct PBj-psi10 [10], pMD.G coding for VSV-G and the appropriate pcDNA3.1 expression plasmid encoding WT Vpx or Vpx T17A for generation of Vpx-containing VLPs. For generation of empty VLPs, pcDNA3.1 was used instead of a Vpx-expressing plasmid as described earlier [9]. For generation of HIV-1 EGFP, the plasmid pHR-CMV-EGFP, the HIV-1 packaging construct pCMVΔR8.9 and pMD.G for generation of HIV-1 particles were used as described before [10]. Production of HIV-1-luc single-cycle reporter particles was performed by transfection of HEK 293T cells with pNL4.3R^+^E^-^ luc3 [45] and pCMV-VSV-G using Lipofectamine 2000 (Invitrogen) according to the manufacturer's instructions. Purification of particles and titration of HIV-1 EGFP was described earlier [10]. The amount of VLPs and HIV-1-luc was determined with the Lenti RT Activity Kit (Cavidi). The VLPs were normalized in comparison to PBj-derived vectors of known infectivity. The amount of VLPs per cells was given as MOI-equivalents (MOIeq). Transduction of THP-1 cells with VLPs was performed with a MOI of 2. Infection of the cells was accomplished with 2.5 ng RT HIV-1-luc two hours after VLP transduction. For single-round infection, monocytes were exposed to HIV-1-EGFP for 4 hours and subjected to flow-cytometry analysis five days post-transduction. For MG132 treatment, the cells were cultured with 50 µM MG132 (Calbiochem) for 30 minutes before transduction.

### PBMC Infection and FACS Analysis

Infections were done with two different CCR5 using HIV-1 viral stocks (SF162, R7/3 EGFP). For analysis of HIV-1 SF162 virus replication, PBMC were infected in triplicate with 0.05 MOI of R5-tropic HIV-1 SF162 and cultured up to day 14. Virus in the supernatant was quantified with the Lenti RT Activity Kit (Cavidi). For FACS analysis of PBMC, the cells were fixed and stained with PE-labelled anti-CD14, FITC-labelled anti-CD3, PE-labelled anti-CCR5 (all BD) and PE-labelled anti-CD4 (DAKO) according to the manufactureŕs instructions. For the infections performed with virus stock HIV-1 R7/3 envYU2-EGFP [31], [32], 2×10^5^ PBMC were infected in triplicate with 0.75 ng or 3.75 ng p24 equivalent for 5 hours. Cells were washed after infection and cultured for 7 days in a 96 well plate. Culture supernatants were collected every day and stored at −80°C. Viral spread was monitored by measuring p24 concentrations in the culture supernatants using a p24 ELISA assay (XpressBio) and by monitoring GFP expression by fluorescent microscopy.

### DNA Transfection and Co-Immunoprecipitation (CoIP) Experiments

293T cells were transiently transfected with the respective plasmids using Lipofectamine 2000 (Invitrogen) according to the manufactureŕs instructions. For transfection of HeLa cells, Fugene 6 (Roche) was used in accordance with the manufacturer's instructions. For CoIP transfected cells were lysed, sonicated and incubated with anti-HA beads (Roche). After 1h incubation at 4 C, the beads were washed with lysis buffer and associated proteins were subjected to SDS-PAGE.

### Immunoblot and Antibodies

Cells were lysed in RIPA buffer and sonicated. When using interferon, monocytes were incubated with IFN-α (ProSpec, cat# Cyt204B or Sigma cat#I4784) 48 hours before lysis. Protein extracts were separated via SDS-PAGE and transferred to a nitrocellulose membrane (GE Healthcare). For detection, anti-HA (Roche), anti-SAMHD1 (abcam), anti-tubulin and anti-GAPDH (all Cell Signaling Technology) were used as primary antibodies. Secondary HRP-conjugated anti-mouse and anti-rabbit antibodies were obtained from GE Healthcare.

### Cell Staining and Microscopy

HeLa cells were seeded on a LabTek chambered coverglass (Nunc) and transfected with expression plasmids for Vpx using Fugene 6 (Roche). After 24 hours, cells were fixed, permeabilized and blocked as described previously [9]. Cells were incubated with anti-HA (Roche) and anti-SAMHD1 (abcam) primary antibodies and stained with secondary Alexa-Fluor 594 anti-rabbit, Alexa-Fluor 488 anti-mouse antibodies (both Invitrogen) and DAPI (Chemicon). Images were acquired with a ZEISS LSM Meta confocal microscope. Live cell imaging of infected and non-infected PBMC from Donor 2 (SAMHD1 -/-) and Donor 4 (SAMHD1 +/+) was performed at the Microscopy Core Facility at MSSM, NY. At day 7 of infection, cells were washed once carefully with warm PBS/1% FBS and stained with anti-human CD14-PE (Clone MEM-15, Abcam, USA) and mouse IgG1-PE (Clone X40, BD Biosciences, USA) for 30 minutes in the 96 well-plate in which the infections were performed. After three washes Hoechst 33342 (10 µM final concentration) stain was added for 30 minutes. Imaging was performed on Olympus IX70 microscope with a live cell imaging system (37°C, 5% CO_2_). Images were acquired with 20x and 40x lenses.

### SAMHD1 Knockdown and Luciferase Reporter Experiments

THP-1 stable cell lines were generated by transduction with lentiviral vectors encoding unspecific shRNA (pLKO-*nontarget*) or shRNA specific to SAMHD1 (shSAMHD1 #1 TRCN0000343807: sequence: CCGGCCCTGAAGAAGATATTTGCTTCTCGAGAAGCAAATATCTTCTTCAGGGTTTTTG); shSAMHD1 #2 TRCN0000343808: sequence CCGGGCCATCATCTTGGAATCCAAACTCGAGTTTGGATTCCAAGATGATGGCTTTTTG) (all Sigma) and selected with puromycin. For HIV-1 luciferase reporter assays, 2.5×10^4^ THP-1 cells were stimulated with 5 ng/ml PMA (Calbiochem) overnight and infected by spin-occulation with HIV-1-luc. Luciferase activity was detected using BriteLite reagent (PerkinElmer) according to the manufacturer's instructions 24 hours post infection.

### Multiplex ELISA

Quantification of IL-6, IP-10, TNF-α, MIP-1β, IFN-α, IFN-γ, RANTES and IL-1β release in supernatants of infected and non-infected PBMC from Donor 2 (SAMHD1 -/-) and Donor 4 (SAMHD1 +/+) was performed using the MILLIPLEX Multi-Analyte Profiling Human Cytokine/Chemokine Kit (Millipore, MA, USA) according to the manufacturer's instructions. Data were analyzed using the Milliplex Analyst software.

### IFN Bioassay

Functional type I interferon was detected using a bioassay previously described [46]. Vero cells were incubated with serial dilutions of culture supernatant of uninfected or HIV-1 infected PBMC for 24 hours and then infected with GFP-encoding Newcastle disease virus (NDV-GFP) (MOI of 1). GFP was measured 18 hours post-infection. A serial dilution of recombinant IFN-β served as positive control and standard.

## Supporting Information

Figure S1 **Vpx binding proteins identified by mass spectrometry.** Prominent SIV-Vpx binding proteins isolated by tandem affinity purification and identified by mass spectrometry (MS/MS) are listed. The individual SwissProt accession numbers and ProteinLynx Global Server 2.3 (PLGS) scores for detection in the UniProt database are given for each protein. Peptides indicate the number of peptides that can be aligned with the respective proteins. The coverage indicates the percentage of protein amino acid sequence that is covered by the peptides assigned to the respective database entry.
(TIF)

Figure S2 **Absence of SAMHD1 expression in cells of AGS patients.** PBMC from healthy donors (Donor 3/4 +/+) or AGS patients homozygous for R164X SAMHD1 (Donor 1/2 -/-) were isolated and infected as described in Figure 3C (left panel). At day 14 post-infection, the cells were lysed and subjected to western blot analysis with the indicated antibodies.
(TIF)

Figure S3 **CD14+ non-infected cells of AGS patient 2 and healthy Donor 4.** A) PBMC from a healthy donor (Donor 4) or an AGS patient with homozygous R164X SAMHD1 mutation (Donor 2) were cultured for seven days. The cells were stained with PE-labeled CD14-targeting antibody and analyzed by live cell fluorescent microscopy (Olympus IX-70, 20x magnification). B) Relative amount of CD14+ cells within cells from Donor 2 (SAMHD1 -/-) and Donor 4 (SAMHD1 +/+) determined by cell counting of 16 and 12 independent microscopy images, respectively, taken after 7 days of cultivation, as exemplarily shown in Figure S3A.
(TIF)

Figure S4 **HIV-1 infection of PBMC from AGS patient 2 and healthy Donor 4 does not induce type I interferon.** Vero cells were incubated with serial dilutions of culture supernatant from day 7 of uninfected (mock) or HIV-1 infected (+ HIV) PBMC of a healthy donor (Donor 4 SAMHD1 +/+) or an AGS patient (Donor 2 SAMHD1 -/-) for 24 hours and were then infected with interferon sensitive GFP-encoding Newcastle disease virus (NDV) with a MOI of 1. GFP was measured 18 hours post-infection by fluorometry. A serial dilution of recombinant IFN-β served as positive control (lower panel, IFN-β), mock addition as negative control (lower panel, mock).
(TIF)
