# Supplementary figures and images for: SAMHD1-Deficient CD14+ Cells from Individuals with Aicardi-Goutières Syndrome Are Highly Susceptible to HIV-1 Infection
Source: PLoS Pathog. 2011 Dec 8;7(12):e1002425. doi: 10.1371/journal.ppat.1002425 (PMC3234228; doi:10.1371/journal.ppat.1002425)

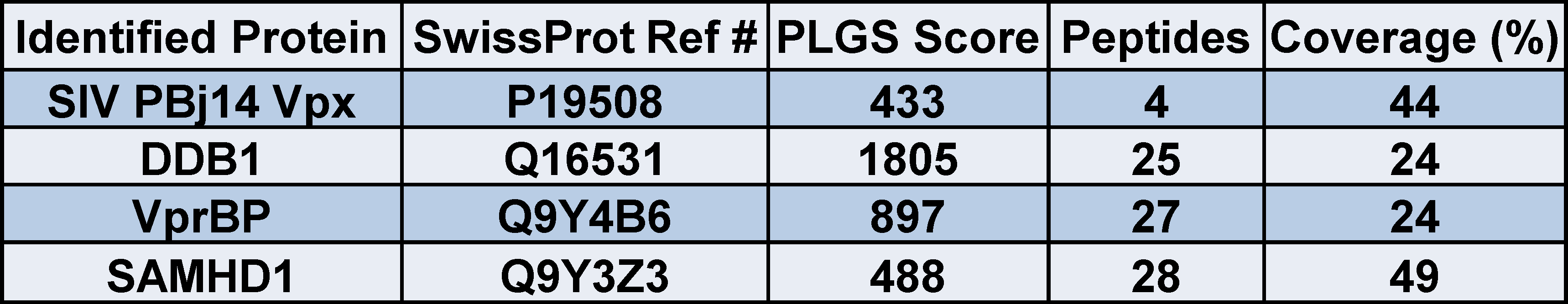

Supplement: Figure S1 — Vpx binding proteins identified by mass spectrometry. Prominent SIV-Vpx binding proteins isolated by tandem affinity purification and identified by mass spectrometry (MS/MS) are listed. The individual SwissProt accession numbers and ProteinLynx Global Server 2.3 (PLGS) scores for detection in the UniProt database are given for each protein. Peptides indicate the number of peptides that can be aligned with the respective proteins. The coverage indicates the percentage of protein amino acid sequence that is covered by the peptides assigned to the respective database entry. (TIF) [file ppat.1002425.s001.tif]

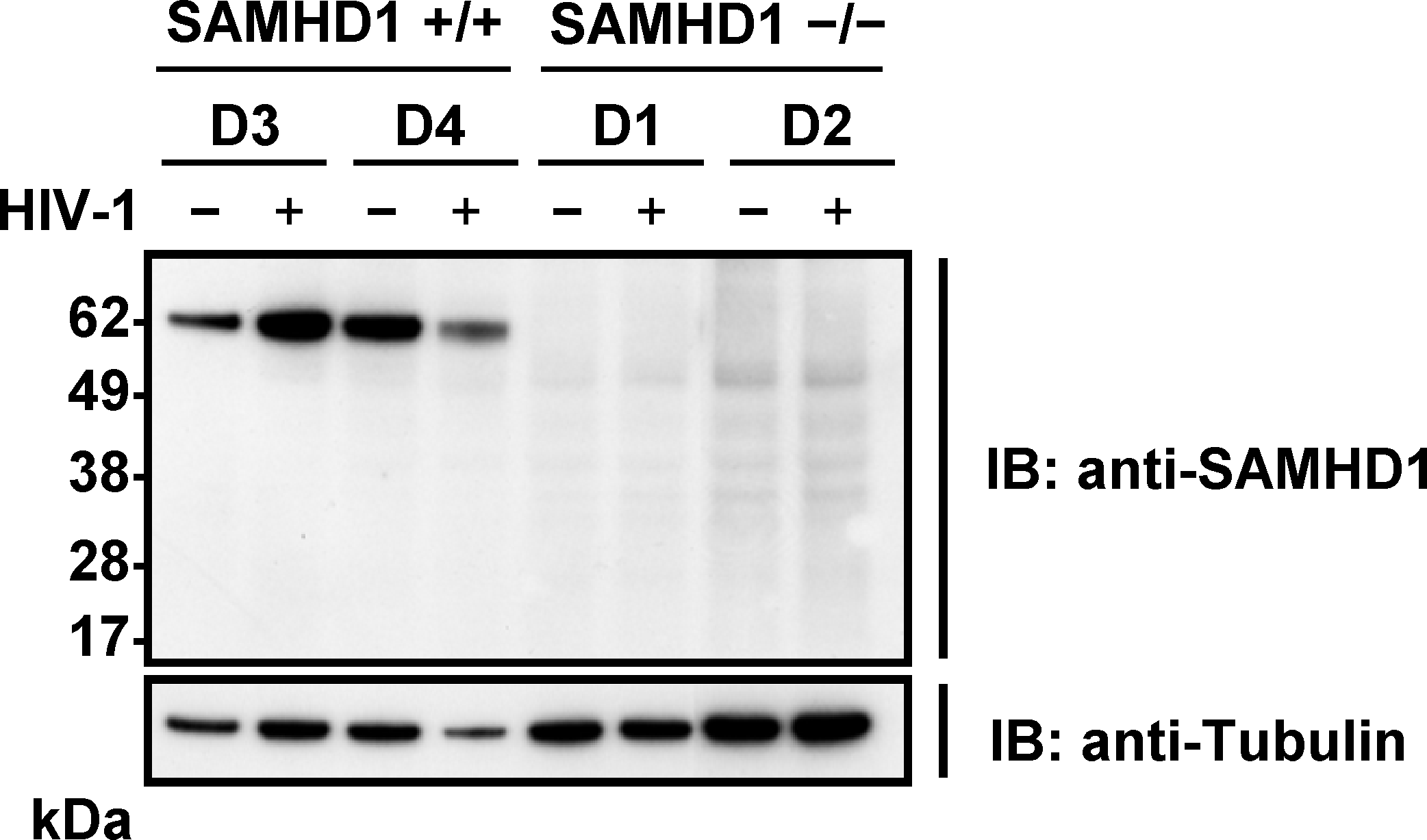

Supplement: Figure S2 — Absence of SAMHD1 expression in cells of AGS patients. PBMC from healthy donors (Donor 3/4 +/+) or AGS patients homozygous for R164X SAMHD1 (Donor 1/2 -/-) were isolated and infected as described in Figure 3C (left panel). At day 14 post-infection, the cells were lysed and subjected to western blot analysis with the indicated antibodies. (TIF) [file ppat.1002425.s002.tif]

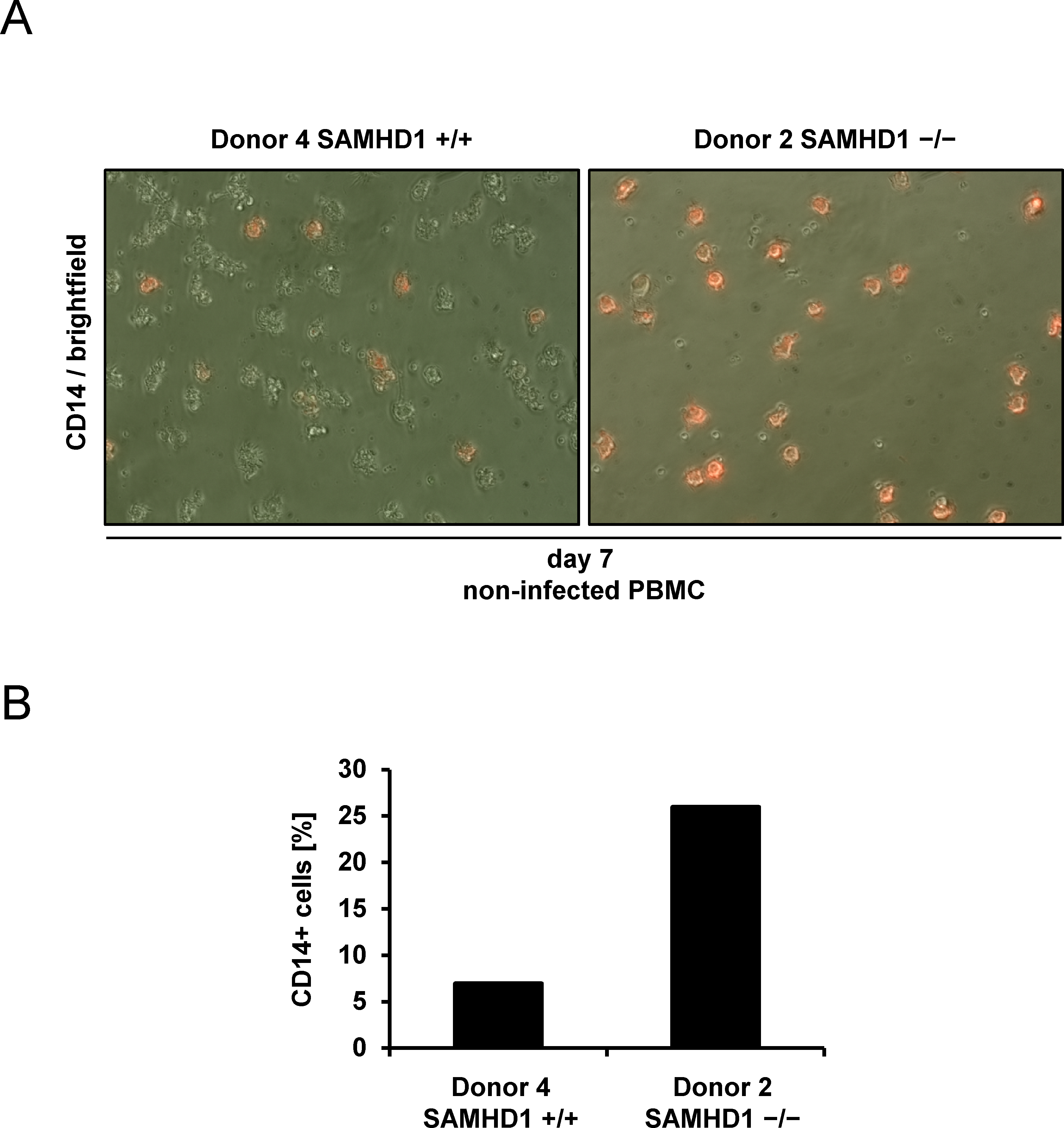

Supplement: Figure S3 — CD14+ non-infected cells of AGS patient 2 and healthy Donor 4. A) PBMC from a healthy donor (Donor 4) or an AGS patient with homozygous R164X SAMHD1 mutation (Donor 2) were cultured for seven days. The cells were stained with PE-labeled CD14-targeting antibody and analyzed by live cell fluorescent microscopy (Olympus IX-70, 20x magnification). B) Relative amount of CD14+ cells within cells from Donor 2 (SAMHD1 -/-) and Donor 4 (SAMHD1 +/+) determined by cell counting of 16 and 12 independent microscopy images, respectively, taken after 7 days of cultivation, as exemplarily shown in Figure S3A. (TIF) [file ppat.1002425.s003.tif]

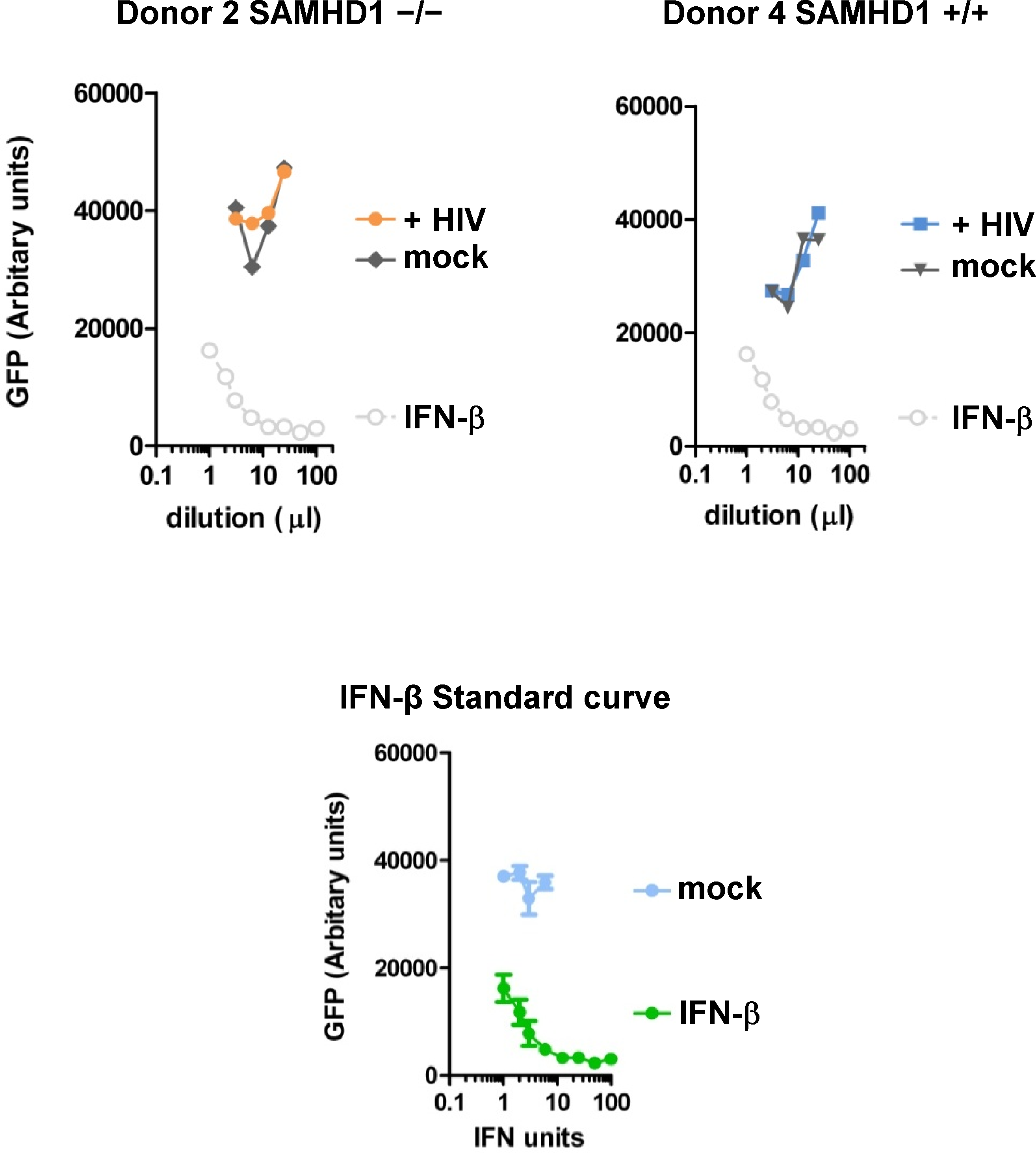

Supplement: Figure S4 — HIV-1 infection of PBMC from AGS patient 2 and healthy Donor 4 does not induce type I interferon. Vero cells were incubated with serial dilutions of culture supernatant from day 7 of uninfected (mock) or HIV-1 infected (+ HIV) PBMC of a healthy donor (Donor 4 SAMHD1 +/+) or an AGS patient (Donor 2 SAMHD1 -/-) for 24 hours and were then infected with interferon sensitive GFP-encoding Newcastle disease virus (NDV) with a MOI of 1. GFP was measured 18 hours post-infection by fluorometry. A serial dilution of recombinant IFN-β served as positive control (lower panel, IFN-β), mock addition as negative control (lower panel, mock). (TIF) [file ppat.1002425.s004.tif]
